# Propylene Glycol Ethers: Widespread Use and Missing Neurotoxicity Testing

**DOI:** 10.3390/toxics14030232

**Published:** 2026-03-09

**Authors:** Nancy B. Hopf, Hélène P. De Luca

**Affiliations:** Unisanté, University Center for Primary Care and Public Health & University of Lausanne, 1010 Lausanne, Switzerland; helene.de-luca@unisante.ch

**Keywords:** glycol ethers, propylene glycol ethers, neurotoxicity, occupational exposure, biomonitoring, chemical risk assessment

## Abstract

Organic solvents are known to affect the nervous system, but neurotoxicity testing is not routinely required for industrial chemicals under current European regulations. Glycol ethers are widely used in consumer and industrial products. They can cross skin and lung barriers, distribute systemically, and penetrate the blood–brain barrier due to their physicochemical properties, while their neurotoxic potential remains poorly characterized. P-series glycol ethers now dominate the European market, making exposure assessment critical for public health. We compiled and integrated data from five authoritative sources to build an inventory of glycol ethers currently used in Europe and performed a structured descriptive analysis of high-volume propylene glycol ether compounds. Six high-volume compounds (≥1000 t/year) were selected for analysis. Production volumes, Swiss product registrations, occupational exposure limits, and product categories were compiled. Propylene glycol methyl ether (PGME) showed the highest tonnage (100,000–1,000,000 t/year) and was present in 9497 registered products, followed by propylene glycol ethyl ether (PGEE) (10,000–100,000 t/year; 1333 products). Paints/coatings and cleaning agents were the most frequent product categories, while additional presence in personal care and indoor-use products was observed. These products may lead to exposure depending on use conditions, such as spraying or inadequate ventilation, which can increase inhalation and skin contact. Their presence in diverse products suggests potential for both occupational and chronic low-level exposures. By providing an integrated overview of market presence, use patterns, and available neurotoxicity evidence for propylene glycol ethers, our findings highlight a critical gap in chemical risk assessment: the absence of neurotoxicity testing despite high production volumes and widespread use. Integrating neurotoxicity endpoints and new approach methodologies into regulatory frameworks is essential to strengthen public health protection.

## 1. Introduction

Glycol ethers are widely used organic solvents with amphiphilic properties that allow them to dissolve both water-soluble and hydrophobic substances [1]. They are essential components in numerous consumer and industrial products, including paints, coatings, cleaning agents, inks, adhesives, and cosmetics [2,3]. The glycol ether family consists of two major subgroups: ethylene glycol ethers and propylene glycol ethers [4].

In 2005, de Ketttenis a representative of the European Chemical Industry Council (CEFIC), emphasized that glycol ethers were among the most studied solvents and could be used safely in consumer and industrial applications when handled according to established guidelines [3]. de Ketttenis argued that media concerns in France had exaggerated risks by generalizing toxicity issues linked to only a few ethylene glycol ethers, such as ethylene glycol methyl ether (EGME) and ethylene glycol ethyl ether (EGEE), to the entire glycol ether family. de Ketttenis also stressed substitution principles, including avoiding risk transfer, ensuring technical feasibility, and maintaining performance, while acknowledging ongoing research and regulatory reviews to confirm safety profiles. Since then, ethylene glycol ethers have been progressively restricted under Registration, Evaluation, Authorization and Restriction of Chemicals (REACH) and Classification, Labelling, Packaging (CLP) Regulations because some were classified as toxic to reproduction (Category 1B) [5,6]. This regulatory action led to their replacement by propylene glycol ethers, which are considered less hazardous and now dominate the global market [7]. Propylene glycol ethers differ chemically from ethylene glycol ethers by having a propylene backbone, which reduces metabolic toxicity and reproductive hazard potential [2].

The substitution of ethylene glycol ethers with P-series propylene glycol ether alternatives has been largely successful for meeting regulatory requirements on endpoints such as reproductive toxicity. However, neurotoxicity, a well-documented risk for many organic solvents, remains largely overlooked in current regulatory frameworks [8,9]. European regulations do not mandate systematic neurotoxicity testing for industrial chemicals unless they are pesticides or structurally suspected to affect the nervous system [5,10]. Under the CLP regulation, the only hazard statement related to neurotoxicity is H336, “May cause drowsiness or dizziness,” which addresses acute exposure [11]. Chronic neurological outcomes such as memory impairment, cognitive decline, or increased risk of neurodegenerative diseases are not considered [12].

This regulatory gap is concerning because glycol ethers share physicochemical pro-perties with other organic solvents known to affect the nervous system [13,14,15]. Solvent exposure has been associated with chronic toxic encephalopathy, sensory deficits, and even neurodegenerative conditions such as Parkinson’s and Alzheimer’s disease [8,16]. Glycol ethers can cross the blood–brain barrier, yet their long-term effects on the human nervous system remain poorly understood [13]. Despite decades of widespread use in consumer and industrial products, biomonitoring studies and epidemiological data on neurological outcomes are scarce [17,18].

Adding to these concerns, recent in vitro studies using human-relevant models have reported structural and functional changes in brain cells exposed to certain propylene glycol ethers at occupationally relevant concentrations [19]. While these findings do not establish definitive risk, they provide mechanistic plausibility and underscore the need for systematic evaluation of neurotoxicity endpoints. Given the large production volumes of propylene glycol ethers and their presence in thousands of products [20], understanding exposure scenarios for workers and consumers is critical for public health.

Our aim was to identify production volumes using and product categories using official registries and suggest potential propylene glycol ethers exposure routes among workers and consumers. Furthermore, we advocate for the systematic inclusion of neurotoxicity endpoints in chemical safety assessments and the adoption of human-relevant new approach methodologies (NAMs), such as advanced in vitro models, to detect early neurotoxic signatures.

## 2. Methods

We compiled glycol ether data from five authoritative sources that provide chemical safety and toxicological data: the European Centre for Ecotoxicology and Toxicology of Chemicals (ECETOC), the European Solvents Industry Group (ESIG), the Hazardous Chemicals and Occupational Diseases database (HAZ-MAP), the International Labour Organization (ILO) International Chemical Safety Cards (ICSCs), and the Institut national de la santé et de la recherche médicale (INSERM). Evidence of neurotoxicity was identified from a broad range of experimental, clinical, and regulatory sources and defined as any adverse effect on the nervous system. Neurotoxicity was considered to encompass clinical and neurobehavioral impairments as well as mechanistic, cellular, or developmental alterations affecting neuronal function, neurotransmission, or nervous system integrity, reported in in vitro, in vivo animal, or human studies [19]. We combined data from these sources and built a comprehensive inventory of glycol ethers used across Europe. We selected six glycol ethers as representative examples based on three criteria: (1) belonging to the propylene glycol ethers; (2) manufactured or imported into the European Union (EU) at high volumes (≥1000 t/year) according to publicly available data from the European Chemicals Agency (ECHA); and (3) either identified as neurotoxic in published literature or regulatory databases, or lacking any neurotoxicity data. We excluded substances without quantitative tonnage information on the ECHA website, acetate derivatives due to similar toxicokinetics and toxicity profiles, and substances for which published toxicological or regulatory assessments consistently reported no neurotoxic effects under the conditions investigated. We extracted data for each selected glycol ether on annual tonnage bands, the number of registered products in Switzerland in 2024, occupational exposure limits (OELs) where available, and the product categories in which these substances are used. We presented the selection process of propylene glycol ethers as a flowchart and the distribution of product categories for each glycol ether as pie charts.

## 3. Results

**Figure 1:**
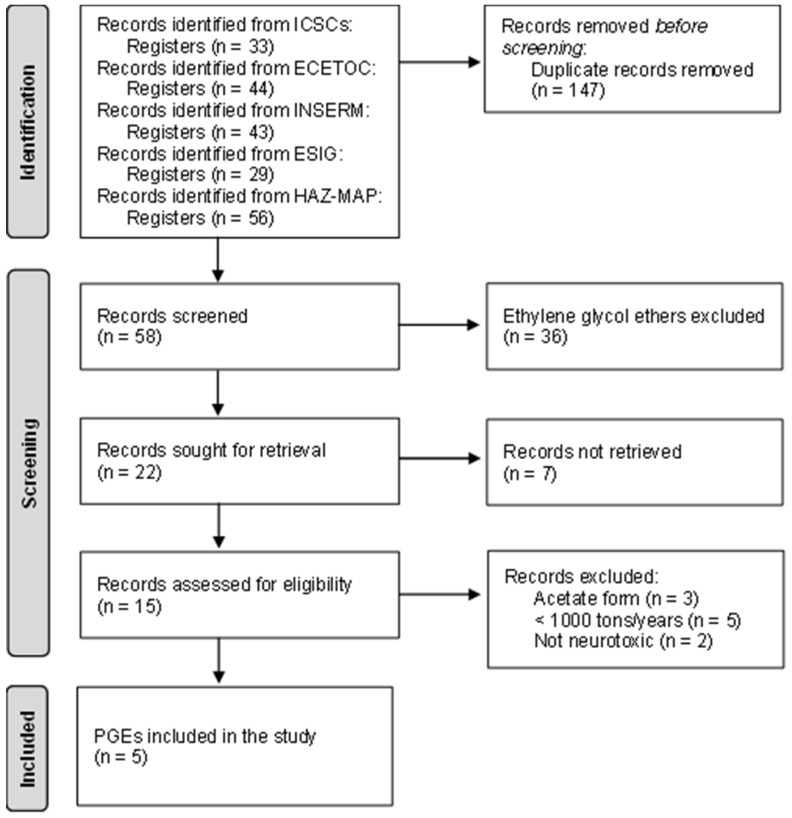
Flowchart illustrating the selection process of the six most used propylene glycol ethers.

We applied three criteria to prioritize substances for evaluation: (1) quantity manufactured and/or imported into the EU per year, (2) evidence of neurotoxicity in published literature or regulatory databases, and (3) number of registered products in Switzerland. Figure 1 shows the flowchart of the selection process.

PGME showed the highest production volume, ranging from 100,000 to 1,000,000 t/year PGME was present in 9497 registered products in Switzerland in 2024. PGEE ranked second in terms of product registration (1333 products) and tonnage (10,000–100,000 t/year). PGBE, PGPE, and PGPhE were produced in smaller quantities (>10,000 t/year for PGBE; 1000–10,000 t/year for PGPE and PGPhE) but remain relevant due to their presence in hundreds of products. DPGBE showed intermediate tonnage (10,000–100,000 t/year) and was registered in 269 products.

Table 1 indicates that neurotoxicity has been documented for PGME, PGEE, PGPE, and PGBE, while no data exist for PGPhE and DPGBE.

Table 2 presents the tonnage for each of the six propylene glycol ethers and the number of registered products by the Federal Office of Public Health (FOPH) containing these solvents. Ingredient lists were not cross-referenced, so individual products may appear multiple times.

### Current Use of Glycol Ethers in Consumer and Industrial Products

**Figure 2:**
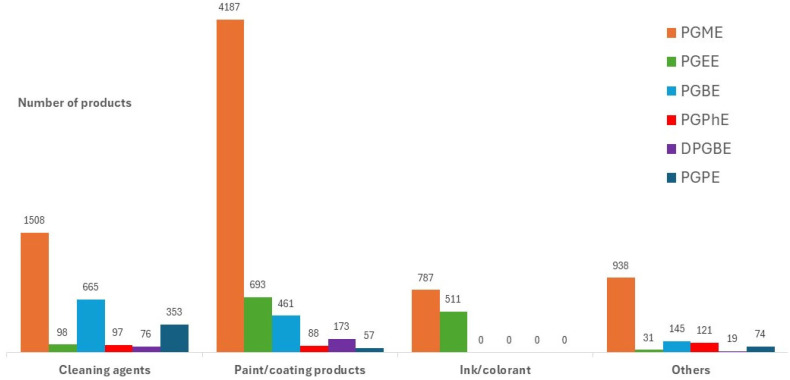
Distribution of product categories for the six selected glycol ethers based on official chemical product declarations.

Figure 2 illustrates the distribution of the six propylene glycol ethers in different product types. Each bar represents one of the six selected propylene glycol ethers.

Glycol ethers are widely present in multiple product categories, confirming their extensive use in both professional and consumer applications. PGME was the most frequently declared propylene glycol ether. It appeared in a broad range of products, including paints and coating agents, cleaning agents, inks and colorants, and personal care products. PGEE and PGBE were also widely used, primarily in paints/coatings and cleaning agents, while PGPE and DPGBE were present in adhesives, sealants, and laboratory chemicals. PGPhE showed a more restricted use profile, mainly in lubricants and hydraulic fluids. Across all substances, paints/coatings and cleaning agents represented the dominant categories. Their extensive use in industrial environments inherently suggests potential occupational exposures for painters, cleaners, and maintenance workers. The presence of glycol ethers in personal care products and indoor-use formulations suggests potential chronic low-level exposure for consumers.

## 4. Discussion

We provide an updated characterization of the current use of propylene glycol ethers in Europe using multiple international databases together with detailed product information from the Swiss market registry. We also highlight a gap in chemical risk assessment, namely, the absence of systematic neurotoxicity testing for propylene glycol ethers. PGME alone accounted for up to one million tons per year in the EU and was present in thousands of registered products. Other propylene glycol ethers such as PGEE, PGPE, and PGBE were widely used in paints, coatings, cleaning agents, and adhesives.

The combination of high production volumes, widespread product use, and absence of OELs highlights a potential for occupational exposure as well as for consumers. Workers in the painting, cleaning, and printing sectors are likely to experience repeated inhalation and skin contact with glycol ethers, while consumers may be exposed chronically through indoor air contamination and personal care products. Both routes are important because propylene glycol ethers are rapidly absorbed after inhalation [38] and dermal uptake [20,39], enabling systemic distribution to all organs, including the brain. Physical tasks, such as cleaning, can further increase uptake and overall dose [40]. Biomonitoring remains the most effective approach to assess total exposure because it accounts for both routes; however, existing studies remain scarce [18]. Moreover, the short apparent urinary elimination half-lives [41], require precise timing of sample collection relative to exposure events to avoid misclassification. Expanded biomonitoring efforts are needed to better characterize human population exposures and strengthen public health protection.

Neurotoxicity data for P-series glycol ethers remain sparse [42]. Recent experimental findings warrant vigilance. Human induced pluripotent stem cell-derived BrainSpheres combined with a multi-omics approach showed that PGBE and its metabolite 2-butoxypropanoic acid (2BPA) induce molecular and cellular alterations affecting nervous system-altered pathways at concentrations relevant to occupational exposure [43]. Similar effects were observed in 3D rat primary mixed brain cell cultures, where PGBE caused neurotoxicity at approximately 3.3 mM [44]. These findings suggest comparable sensitivity in rats and humans. However, the specific molecular pathways and key initiating events underlying these effects remain unknown, and it is unclear whether parent compounds and their β-isomer-derived metabolites act through shared or distinct mechanisms within the CNS. Although the number of studies is limited, the evidence indicates that PGEs can produce neurotoxic effects that remain largely uncharacterized.

Propylene glycol ethers are sold as mixtures of two isomers: an α-isomer (secondary alcohol) and a β-isomer (primary alcohol). The α-isomers are metabolized to propylene glycol or conjugates (sulfate/glucuronide), considered non-toxic [44]. In contrast, β-isomers undergo metabolism similar to ethylene glycol ethers, forming alkoxypropionic acids. This was observed in rodents in vivo [45] and in human brain cells in vitro [43]. Currently, only PGME is regulated to contain <5% β-isomer due to concerns that its metabolite may exert toxicological effects similar to propylene glycol ethers. No comparable restriction exists for other propylene glycol ethers, and the β-isomer fraction in technical-grade products is typically unspecified.

The amphiphilic properties of propylene glycol ethers make them effective cleaning agents. They are able to dissolve both fats and water. This may also underlie their potential to disrupt biological membranes. This lipid-solubilizing capacity can interfere with membrane integrity and signaling, particularly within lipid rafts enriched in glycosphingolipids, gangliosides, and cholesterol [19,46]. Early and pronounced alterations in lipid profiles after exposure to PGBE and its β-isomer metabolite 2-butylpropionic acid suggest that membrane disruption may be an initiating event in neurotoxicity. PGME and PGBE are readily absorbed into the bloodstream following inhalation, the primary route of exposure [38]. They can reach the brain under typical workplace conditions, as demonstrated by toxicokinetic modeling and blood–brain barrier permeability studies [43,44,47]. Given PGBE’s greater lipophilicity and higher permeability compared to PGME, its brain concentrations are expected to be even higher. Notably, symptoms such as headaches have been reported in workers exposed to PGME [21], and PGBE has been associated with more pronounced neurotoxicity-related outcomes than PGME and EGME in experimental models [44]. Importantly, occupational and consumer exposures typically involve mixtures or formulations rather than single substances. Human toxicokinetic studies conducted under controlled mixed-exposure conditions show that PGME and PGBE are absorbed and eliminated concurrently [41]. Such findings illustrate the limitations of substance-by-substance hazard characterization for solvents used in combination. Commercial formulations and mixtures, however, are under-represented in public toxicological databases, limiting interpretation of actual potential for neurotoxicity.

Collectively, these findings indicate that the same chemical properties valued for industrial cleaning may also contribute to neurotoxic mechanisms, reinforcing biological plausibility for human health concerns. We recommend systematic neurotoxicity testing to be integrated into chemical safety assessments for glycol ethers and other organic solvents. The fragmented neurotoxicity information in public databases highlights the value of integrating new approach methodologies (NAMs) into regulatory frameworks such as CLP, REACH, and OEL settings. Mechanistic patterns emerging from existing evidence (membrane perturbation, lipid dysregulation, and central nervous system exposure) correspond to early key events represented in several neurotoxicity adverse outcome pathways (AOPs). In this context, in vitro and in silico NAMs could be used within integrated approaches to testing and assessment (IATA). Such approaches support hazard identification, substance prioritization, and read-across among structurally related glycol ethers. While NAMs are not currently sufficient as standalone evidence for regulatory classification, their use could strengthen weight-of-evidence evaluations and guide targeted data generation. Occupational and consumer exposures often involve solvent mixtures or formulations. These are underrepresented in public datasets, limiting the interpretation of real-world neurotoxicity. NAM-based screening of formulations could provide pragmatic, formulation-level signals to guide targeted testing.

Limitations exist for all studies, including ours. This study is constrained by the fragmented nature of neurotoxicity data, the absence of harmonized definitions and testing requirements, and the limited availability of studies for P-series glycol ethers. Exclusion of substances based on consistently negative toxicological or regulatory assessments may bias the selection toward less extensively characterized chemicals; however, this criterion was applied for scoping feasibility and should not be interpreted as evidence of the safety of the excluded substances. An inclusive hazard-identification approach was therefore adopted, recognizing that data scarcity and geographic scope (Swiss regulatory sources) limit definitive conclusions but highlight persisting regulatory gaps, even in stringent frameworks where neurotoxicity is not systematically assessed.

## 5. Conclusions

The widespread use of propylene glycol ethers, combined with emerging evidence of neurotoxic potential, highlights a gap in current chemical risk assessment. Neurotoxicity testing is not systematically required under existing regulatory frameworks, despite their ability to reach the brain under typical exposure conditions. Incorporating neurotoxicity testing into regulatory assessments with NAMs could help with early detection of neurotoxic substances and strengthen public health protection.

## Figures and Tables

**Table 1:** Neurotoxicity data summarized for the propylene glycol ethers.

Compound	Endpoint	Exposure			Species	References
		Route	Dose	Duration		
PGME *	CNS toxicity	Inhalation	1000 ppm	Variable	Humans	[21]
	Sedative effects	Inhalation	3000 ppm	13 weeks	Rats	[22]
	Sedative effects	Inhalation	3000 ppm	13 weeks	Rabbits	[22]
	Transient sedation	Inhalation	3000 ppm	1 week	Rats	[23]
	Transient sedation	Inhalation	3000 ppm	1 week	Mice	[23]
	CNS depression	Inhalation	3000 ppm	Variable	Rats	[24]
	CNS depression	Inhalation	3000 ppm	Variable	Mice	[24]
	Dizziness	-	10,000 ppm	-	Rats	[25]
	CNS depression	Inhalation	3000 ppm	13 weeks	Rats	[26]
	CNS depression	Inhalation	3000 ppm	13 weeks	Rabbits	[26]
**DPGME**	No effect	Inhalation	200 ppm	13 weeks	Rats	[27]
	No effect	Inhalation	200 ppm	13 weeks	Rabbits	[27]
	CNS effects	-	-	-	-	[28]
	Mild narcosis	Inhalation	-	-	Rats	[25]
	CNS depression	Oral	-	-	Rats	[25]
	No effect	Inhalation	200 ppm	13 weeks	Rats	[29]
	No effect	Inhalation	200 ppm	13 weeks	Rabbits	[29]
	No neurotoxicity	-	-	-	-	[30]
**TPGME**	No systemic effects	Inhalation	-	7 h	Rats	[25]
	CNS depression	Oral	-	-	Dogs	[31]
	Low effects	Inhalation	-	-	Rats	[32]
PGEE *	CNS depression	Inhalation	10,000 ppm	4 h	Rats	[33]
**PGPE**	Narcosis	Oral	-	-	Rats	[34]
	Narcosis	Oral	-	-	Rabbits	[34]
	CNS effects	-	-	-	-	[35]
**PGBE**	CNS depression	Oral	-	-	Rats	[36]
**DPGBE**	-	-	-	-	-	N.A.
**PGPhE**	Anaesthetic effects	-	-	-	Gastropods	[37]

* Hazard statement H336, N.A. = not available.

**Table 2:** Selection criteria for six glycol ethers: tonnage manufactured/imported in the EU, evidence of neurotoxicity, number of registered products in Switzerland, and occupational exposure limits (OELs, set by SUVA).

Solvent	CAS #	Quantity Manufactured and/or Imported in the EU (t/Year)	Evidence of Neurotoxicity	Registered Products (FOPH)	OEL (SUVA)
PGME	107-98-2	100,000–1,000,000	Present (+)	9497	100 ppm
PGBE	5131-66-8	>10,000	Present	1271	N.A.
PGEE	1569-02-4	10,000–100,000	Present	1333	50 ppm
PGPE	1569-01-3	1000–10,000	Present	484	N.A.
PGPhE	770-35-4	1000–10,000	Never tested	306	N.A.
DPGBE	29911-28-2	10,000–100,000	Never tested	269	N.A.

N.A. = not available.

## Data Availability

The research data used are from the public domain, except the analysis of the Swiss market, which is confidential.
